# Recent advances in the therapeutic applications of selenium nanoparticles

**DOI:** 10.1007/s11033-024-09598-z

**Published:** 2024-05-25

**Authors:** Jeba Ajgar Ansari, Jonaid Ahmad Malik, Sakeel Ahmed, Muntaha Manzoor, Nafees Ahemad, Sirajudheen Anwar

**Affiliations:** 1 Department of Pharmaceutics, Government College of Pharmacy, Dr. Babasaheb Ambedkar Marathwada University, (BAMU, Aurangabad), India; 2 Department of Biomedical Engineering, Indian Institute of Technology Ropar, Rupnagar, India; 3 Department of Pharmacology and Toxicology, National Institute of Pharmaceutical Education and Research, Ahmedabad, Gujarat India; 4 Department of Clinical Pharmacology, Sher - i - Kashmir Institute of Medical Sciences, Soura, Srinagar India; 5 School of Pharmacy, Monash University Malaysia, Jalan Lagoon Selatan, Bandar Sunway, Petaling Jaya, Selangor, DE 47500 Malaysia; 6 Department of Pharmacology & Toxicology, College of Pharmacy, University of Hail, Hail, Saudi Arabia

**Keywords:** Selenium, Nutrient, Nanoparticles, Therapeutic application, Clinical translation

## Abstract

Selenium nanoparticles (SeNPs) are an appealing carrier for the targeted delivery. The selenium nanoparticles are gaining global attention because of the potential therapeutic applications in several diseases e.g., rheumatoid arthritis (RA), inflammatory bowel disease (IBD), asthma, liver, and various autoimmune disorders like psoriasis, cancer, diabetes, and a variety of infectious diseases. Despite the fact still there is no recent literature that summarises the therapeutic applications of SeNPs. There are some challenges that need to be addressed like finding targets for SeNPs in various diseases, and the various functionalization techniques utilized to increase SeNP’s stability while facilitating wide drug-loaded SeNP distribution to tumor areas and preventing off-target impacts need to focus on understanding more about the therapeutic aspects for better understanding the science behind it. Keeping that in mind we have focused on this gap and try to summarize all recent key targeted therapies for SeNPs in cancer treatment and the numerous functionalization strategies. We have also focused on recent advancements in SeNP functionalization methodologies and mechanisms for biomedical applications, particularly in anticancer, anti-inflammatory, and anti-infection therapeutics. Based on our observation we found that SeNPs could potentially be useful in suppressing viral epidemics, like the ongoing COVID-19 pandemic, in complement to their antibacterial and antiparasitic uses. SeNPs are significant nanoplatforms with numerous desirable properties for clinical translation.

**Graphical Abstract:**

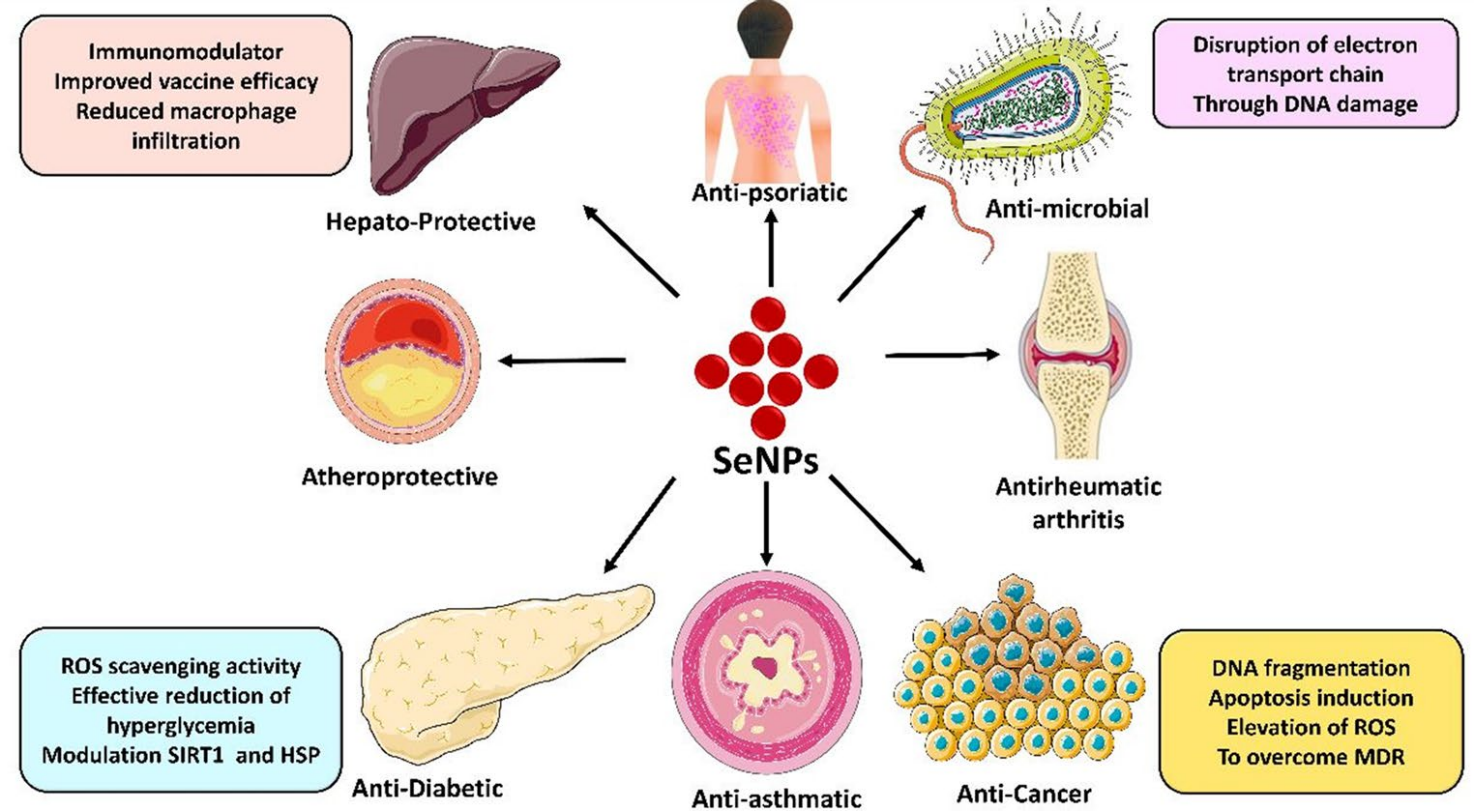

## Introduction

Se is an essential trace element with pleiotropic pharmacological activities, while Se nanoparticles (SeNPs) retain biological properties in the formulated form of nanoparticles for therapeutic applications. Selenium is a vital nutrient that plays a key role in different biological processes in the body and has various therapeutic effects. Selenocysteine (Selenium) is found in 25 mammalian metabolic enzymes and is a preventive factor in various diseases, including hypercholesterolemia, cancer, and cardiovascular disease [1]. Se is an essential nutrient with potential therapeutic and pharmacological effects through its assimilation into selenoproteins, a trait which other metal nanoparticles lack. Amongst the most significant ways that selenoproteins alter the action of protein kinases, phosphatases, and regulatory genes like NF-κB is by thiol oxidation of signaling proteins [2, 3].

SeNPs outperform selenite (SeO3) and selenate (SeO4) molecules in anticancer, nontoxicity, and biocompatibility. The invasion of apoptotic cell death and cell cycle arrest (CCA), which ultimately leads to blocking other mechanisms, are the primary mechanisms behind SeNPs’ anticancer properties. SeNPs have the potential to open up new routine techniques for providing treatment to conditions like cancer, and this assessment explains how such nano-measured drugs could become a promising approach in chemotherapeutics [4].

Because of the functionalization of SeNPs with specific targeting ligands, they can deliver combinations of therapies to the targeted cells. SeNPs have shown potential in various disorders, including diabetes, Alzheimer’s disease, inflammatory diseases including RA, atherosclerosis, cancer, several bacterial, fungal, and viral infections, and stroke [5].

The global market of metal nanoparticles in 2021 was 2.4 billion US dollars and is predicted to rise to 4.2 billion US dollars by 2026 with an ever increasing demand in healthcare and pharmaceutical applications [6].

### The unique characteristics of SeNPs

Selenium nanoparticles (SeNPs) are gaining popularity due to their high permeability and nontoxicity. Because of its several advantages, it could be used in the targeted delivery of drugs as an anticancer agent, as an antioxidant against several drug-induced toxicities, and as an anti-diabetic and anti-inflammatory agent [1]. SeNPs have excellent bioactivity; however, poor cellular intake is a drawback. Functionalization of specific ligands on the external surface of NPs has been tried to solve this problem. Surface sealing agents regulate the size, durability, cancer specificity, cellular uptake of SeNPs, and bioavailability and bioactivity. SeNPs could also deliver differently charged macromolecules and antitumor agents at a nanoscale. SeNPs exhibit high antitumor activity and lesser safety concerns when compared to other Se forms. In vitro, SeNPs consume radicals in a size-dependent approach (5–200 nm). Small-sized (5–15 nm) SeNPs have a greater radical-scavenging ability and can inhibit DNA damage [7, 8].

SeNPs constitute an attractive carrier platform to ferry various drugs to the site of action. Although the genomic impacts of SeNPs and their mode of action on the expression of genes are still uncertain, selenium-based diagnostics may be used in the future. Nanomaterials could monitor epigenetic variables that have been intensively researched and may have a critical role in the early stages of cancer diagnosis [9]. The other unique features of Se compared to other metal NPs are that it is colorless and non-toxic, which solely depends on its oxidation status. It is present in several oxidation forms, such as selenite (+ 4) and selenate (+ 6), and selenite (+ 4) is a latent composite for antimicrobial action. It acts as a redox center for many antioxidant proteins or enzymes [10].

### Synthesis of selenium nanoparticles

Developing and constructing smart NPs for clinical translation has been the ultimate goal of precision nanomedicine. However, synthesizing NPs free of hazardous or harmful materials is difficult, particularly for nanomedicine. This search has influenced the development of various SeNP synthesis methods [11]. In general, physical, chemical, and biological techniques can be used to generate selenium nanoparticles. The organically produced SeNPs, however, show improved compatibility with human organs and tissues. Numerous researchers have investigated how their use in biological systems is affected by their size, shape, and fabrication technique [12]. The biosynthesis of SeNP was described in a study by introducing BSA to the redox system of sodium selenite and glutathione. In mice, the synthesized SeNP was less hazardous than sodium selenite in toxicities [13].

There are various methods to synthesize the SeNPs segregated into 3 major methods: biological, physical, and chemical. The physical method includes vapor deposition, solvothermal, laser ablation, and hydrothermal technologies. The pulsed laser method is advantageous because it is easy to collect the NPs via centrifugation and stability [10].

The chemical method reduces selenium into precursors, the most utilized method for producing SeNPs. Ionic liquid 1-ethyl-3-methylimidazolium thiocyanate, ascorbic acid, glucose, glutathione, sodium meta sulfite, fructose, and cysteine are the reducing agents that are being used in the preparation of SeNPs, in addition carboxymethyl cellulose (CMC), the water-soluble polymers and bovine serum albumin (BSA) are utilized as the stabling agents to keep them in segregated mode or the prevention of aggregation [10].

The biological method includes the use of biological sources like viruses, bacteria, fungi, algae, plants, and other sources like proteins. The plants have been used in the preparation of SeNPs. Biological waste, such as fruit peels, is also used to synthesize SeNPs. The plant-based secondary metabolites, such as saponin, flavonoids, polysaccharides, alkaloids, etc., are used as reducing and stabilizing agents [10].

New ways are now being applied for the hybrid synthesis of selenium nanoparticles for more efficient delivery and targeting, like in the case of cancer, huge challenges are there regarding drug delivery. One example is the preparation of hybrid endosomes-BSA (NISM-B) hybridized SeNPs (NISM-B@SeNPs) to target cancer, showing remarkable therapeutic efficacy against cancer cell lines [14].

### Present standing of selenium nanoparticles

SeNPs are practical techniques in contemporary biomedical research with extraordinary capabilities as prospective treatments, in line with the fascinating possibilities provided by nanotechnology in diagnostics, management, and disease prevention. Biogenic SeNPs are gaining much attention due to the demand for inexpensive, uncomplicated, high-throughput, environmentally acceptable biomedical agents that can also be used for theranostic applications and have few detrimental consequences. Seeing as their selective and efficient accumulation in tumors via the EPR effect, SeNPs have shown considerable preclinical potential in cancer diagnosis and gene and medication delivery. SeNPs are a strong choice for advanced-stage clinical research since they have low toxicity and outstanding biocompatibility, but there is a shortfall in research on their potential in clinical settings [11]. Se nanoparticle-based diagnosis and therapy are in their early stages and preparing to progress into clinical trials.

## Role of senps in several diseases

**Fig. 1:**
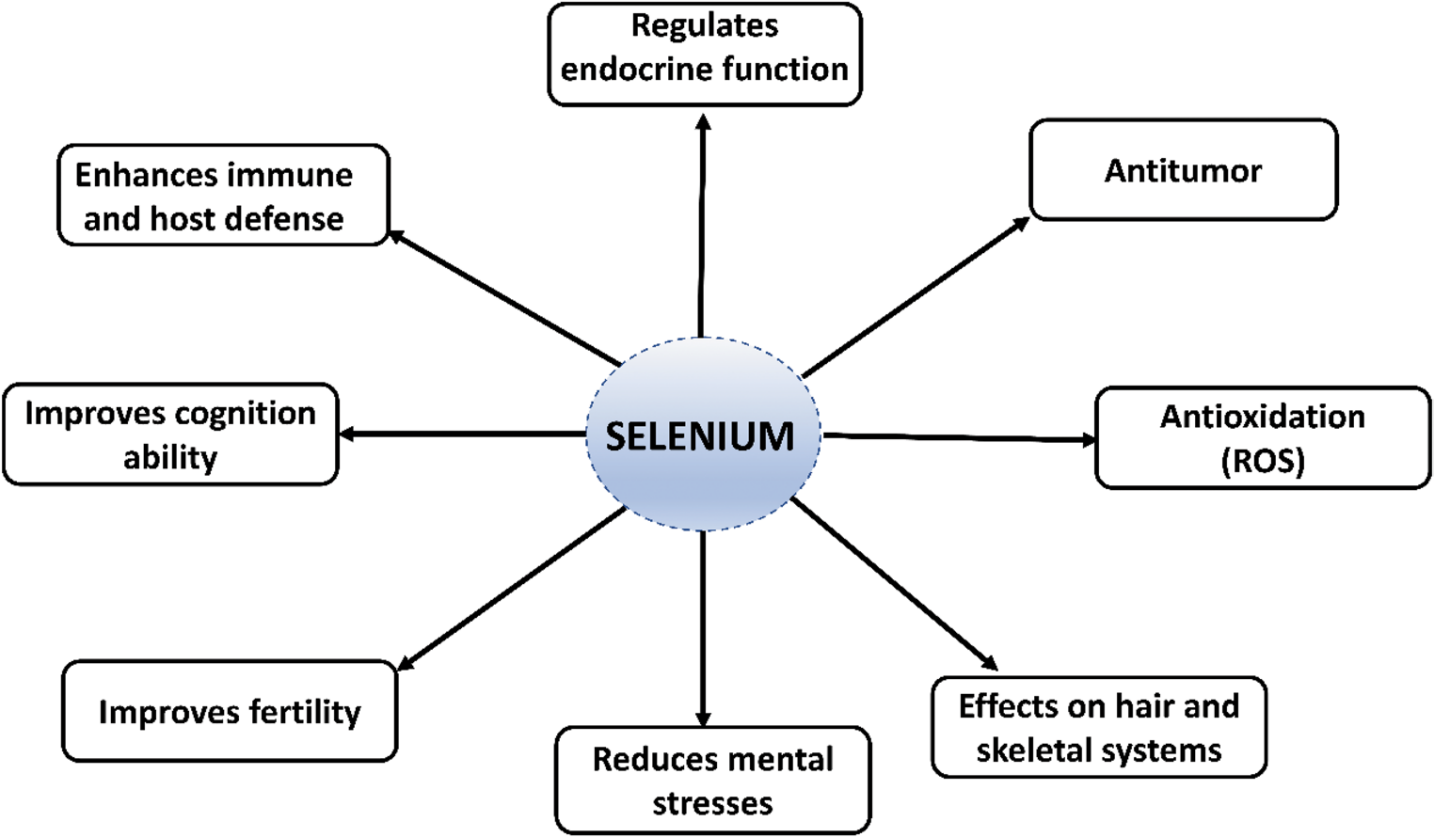
Physiological functions of selenium

SeNPs are becoming prominent in the clinical arena because they can potentially prevent infectious diseases and cancer treatments [15]. SeNPs are considered an effective approach for delivering selenium in biological systems because of their safety and efficacy [16]. This paper will discuss the mechanism and role of various SeNPs that are mentioned for several disease conditions and viral infections. The demonstration of various physiological functions performed by selenium nanoparticles is depicted in Fig. 1.

### Inflammatory diseases

**Fig. 2:**
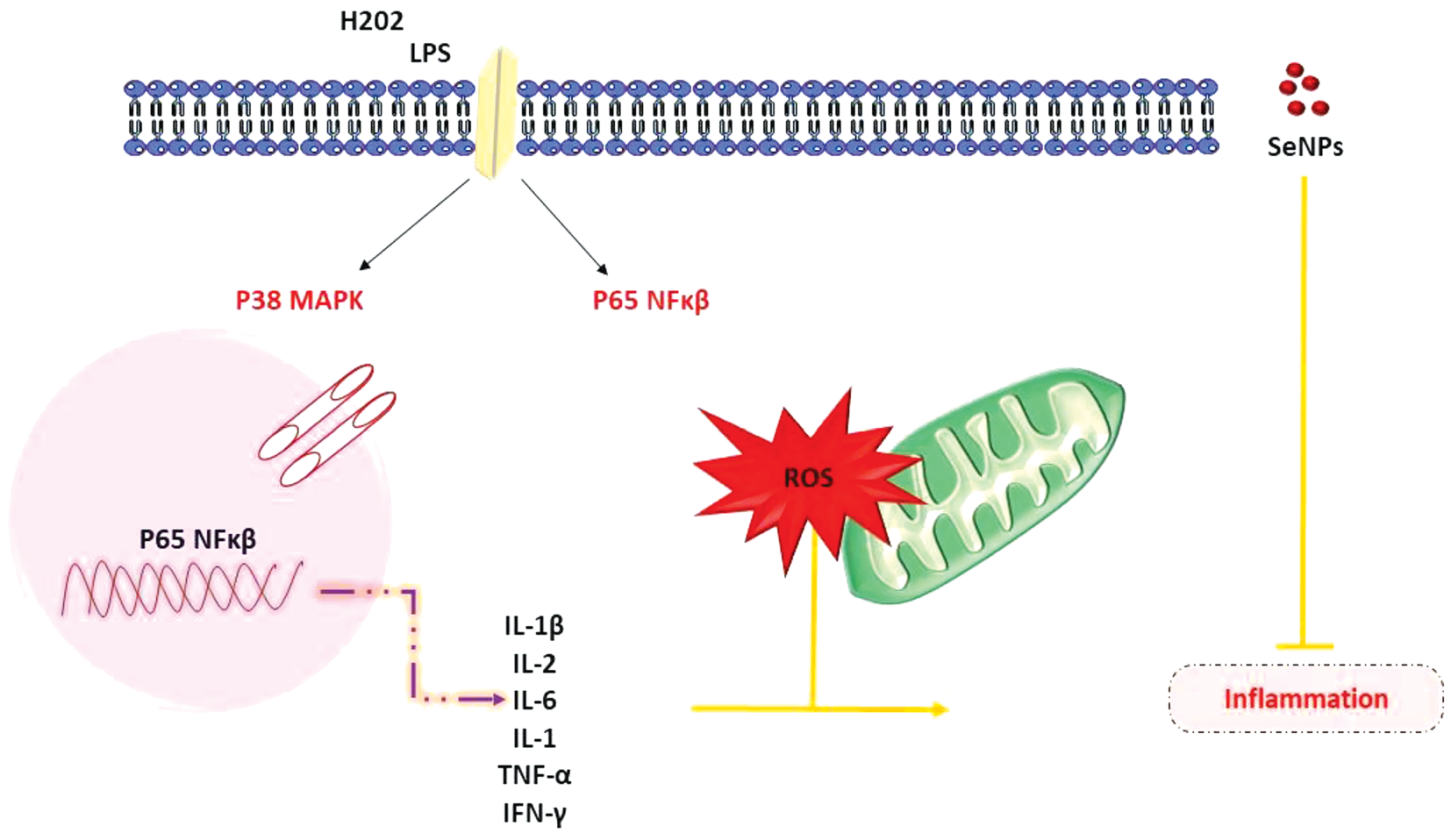
Anti-inflammatory effects of SeNPs. SeNPs effectively disrupt the immune reaction induced by H2O2 or LPS. SeNPs have been found to scavenge ROS due to their antioxidant properties. Activation of the p38 MAPKs and NF-κB has been identified as a mechanism of anti-inflammatory effect, reducing inflammatory cytokine generation [8]

According to a report, active-form functionalized SeNPs may modulate selenoproteins to improve cancer chemotherapy using NK and CI-NK cells over different malignancies. As a result, functionalized SeNPs could become a successful treatment solution for various disorders, and some research groups have been especially interested in SeNPs’ therapeutic efficacy in inflammatory disorders [17–19]. The demonstration of the anti-inflammatory activity of SeNPs in various inflammatory conditions is depicted in Fig. 2.

#### Inflammatory bowel disease

IBD is one of the most challenging disorders to manage, requiring complicated maintenance and carry procedures. IBD affects approximately 1 million population in the US and 2.5 million individuals in Europe. Selenium has been linked to anti-inflammatory characteristics [20]. Selenium insufficiency is frequent among CD and UC individuals. Individuals with IBD, specifically Crohn’s disease, have reduced selenoprotein concentrations. Selenium can increase the protection of gut microbiota in IBD patients. Selenium could be the appropriate alternative for both short-term and long-term treatments in IBD [21].

The nanoparticulated delivery system reduces drug toxicity, improves drug toxicity, improves therapeutic targets, and extends therapeutic efficacy. Selenium nanoparticles were studied in the context of artificial colitis. In DSS-induced colitis, selenium NP could alter the chronic inflammation. The NP inhibited NF-κB expression and reduced the synthesis of IL6 and TNF-α. In a rat model of TNBS-induced colitis, other selenium NP reduced the severity of the damage. Immunomodulating drugs like corticosteroids and anti-TNF medications are used to reduce the amplification of these cytokines, which is the basis of unmanaged IBD [21].

**Fig. 3:**
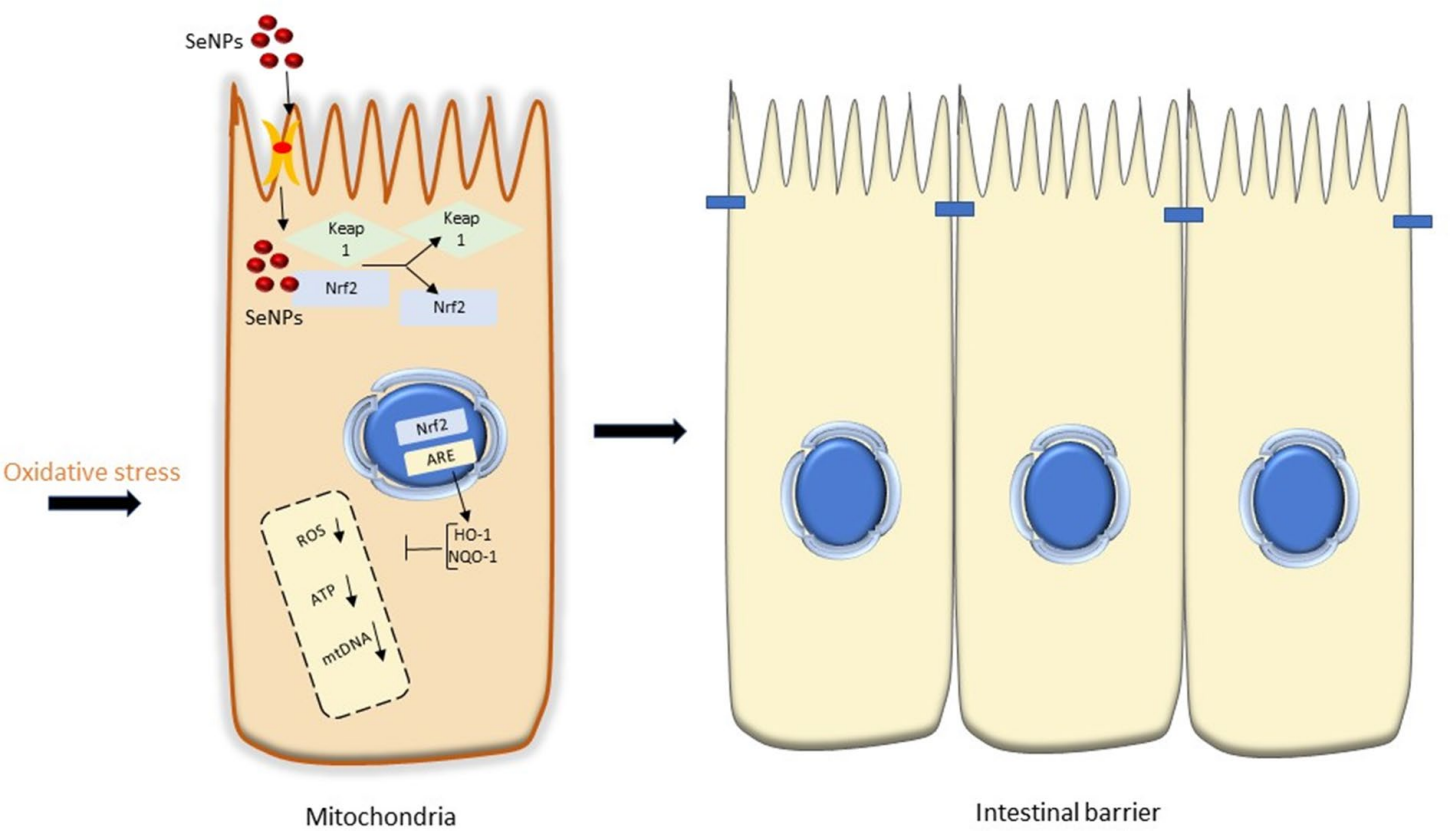
Demonstrate the proposed mechanism of SeNPs that act by protecting the function of the intestinal epithelial barrier against (IEB) oxidative stress. SeNPs protect the function of the IEB from oxidative stress. Nrf2 is detached from the Keap1-Nrf2 complex and transferred into the nucleus when SeNPs are delivered into IE cells, which connect to ARE. With the induction of ARE, the antioxidant enzymes HO-1 and NQO-1 are activated, which work together to suppress ROS generation, weaken MMP depolarization, and increase mitochondrial DNA copy number. In conclusion, SeNPs preserve the IEB from oxidative damage by reducing ROS-related mitochondrial damage through the Nrf2 signaling pathway during oxidative stress [22]

In a report by Xu et al. by mitigating ROS-related mitochondrial dysregulation through the Nrf2 signaling pathway, biogenic SeNPs produced by L. casei ATCC 393 may preserve the intestinal epithelial barrier integrity from oxidative stress. SeNPs enhanced the protein expression of Nrf2, HO-1, and NQO-1. Nrf2 exits in the form of the Nrf2 Keap1 complex. This complex enters the nucleus and acts as an endogenous antioxidant defense system. Furthermore, SeNPs reduced the oxidative stress-induced degradation of mitochondrial structures. SeNPs’ regulating influence on cytoplasmic ROS generation was eliminated by an Nrf2 inhibitor (ML385). By addressing mitochondria, biogenic SeNPs are the popular proposition for a potential Se supplement therapy in reducing oxidative stress-related bowel illnesses, including IBD [22]. The demonstration of the mechanism of SeNPs in managing oxidative stress in IBD is depicted in Fig. 3.

#### Ulcerative colitis (UC)

UC seems to be a recurrent chronic condition that can proceed from undetected minor inflammation to severe colorectal inflammation, resulting in irreversible fibrosis and muscle atrophy. Oxidative stress caused by UC produces structural destruction to lipids, DNA, and peptides, leading to a variety of diseases. In colitis, enzyme indicators of intestinal injury such as ALP and MPO are also high. IL-10 and TNF-α are modulatory cytokines that play a critical role in the etiology and progression of immune and inflammatory responses. Because of their Sgreat accessibility and various biological effects. Selenium in the form of NPs has been shown to have biological effects such as decreasing inflammation by downregulating mRNA synthesis of pro-inflammatory mediators such as inducible-NO-synthase, IL-1, and TNF-α. In a study by Prateek et al., He compared the potential of SeNP and Dexamethasone in Acetic acid-induced UC in male Wistar rats. The SeNPs showed anti-colitis actions in various groups by decreasing the effect of IL-1 and TNF-α in a concentration-dependent manner [23].

Selenium intake might delay the onset of UC by activating genes involved in regulating the immune system. Antioxidant defence components, inflammation, and cell death are all regulated by SIRT1 and are responsible for enhancing the maintenance of inflammatory cells in colitis. According to many clinical and preclinical research, SIRT1 is decreased in UC patients or IBD models, and administration with SIRT1 inducers significantly reduces colitis severity. SIRT1 induce PGC-1α [24–26]. SeNPs, particularly when coated with natural biological components, have anti-inflammatory properties and are low in toxicity. Chenghui Zhu and colleagues investigated the therapeutic benefits of SeNPs coated with ULP in mice with acute colitis. SeNPs prevented upregulation by reducing the nuclear translocation of NF-κB, which is responsible for developing these pro-inflammatory mediators. ULP-SeNPs could be a great choice for further research as a possible therapeutic material for UC and other inflammatory conditions [27, 28].

Hui Yang et al. developed MRO with their most effective anti-inflammatory properties. They used them to enhance the properties SeNPs, intending to resolve inflammation in UC by speeding up macrophage remodeling. MRO-SeNPs successfully reduced colitis by reducing pro-inflammatory mediators such as IL-1ß, IL-6, TNF-α, IL-12, and MCP-1 in colon tissues and exerting significant antioxidant activity. Lastly, MRO-SeNPs showed therapeutic activity by encouraging macrophage remodeling, which resulted in the remission of inflammation [19].

PGs are naturally occurring bacterial components that have antioxidant and immunosuppressive activities. Kassab et al., in the present report, demonstrated the potential preventive effect of PGs coupled with selenium nanoparticles (PGs-SeNPs) against acetic acid-induced UC in rats. In the colon tissue, overexpression of Nrf2 and its downstream antioxidants and reduced pro-oxidants [ROS, carbonyl protein, MDA, iNOS, and NO] showed that PGs-SeNPs enhanced colonic antioxidant capacity and stopped peroxidation attacks. Lowering pro-apoptotic factors [Bax and caspase-3] and raising anti-apoptotic protein, Bcl2, PGs-SeNPs prevented intestinal cells from dying by blocking the apoptotic pathway. Due to its powerful anti-inflammatory, antioxidant, and anti-apoptotic properties, PGs-SeNPs might be employed as an alternate anti-colitic strategy [29].

#### Crohn’S disease (CD)

CD is identified by severe intestine inflammation, and several factors included in its etiology, involve oxidative stress. Certain elements, such as Se, are lacking in CD patients during the disorder’s operative and relapse periods. In a reported study, individuals with CD had more GPx1 activation than normal, which could be described by a homeostatic mechanism activated in response to rising ROS generation due to intestinal obstruction. Furthermore, GPx1 is selectively expressed throughout the post-stress restoration and needs lower selenium for optimum levels comparable to SepP [30].

Yue Chen et al. explained the activity of SeNPs on the K88-induced intestinal barrier caused by *E coli*. In the selected model, oral delivery of *L. lactis* NZ9000-SeNPs elevated the number of goblet cells in the ileum, decreased serum and ileal IL-1, TNF-α, and IFN-γ, and elevated TRx and GPx actions. These results demonstrated that SeNPs could be a promising and effective strategy for managing a variety of inflammatory diseases [31].

#### Psoriasis

The several treatment choices for psoriasis are linked to various side effects. To get around this, a nano-formulation is being developed. Se is a vital element that plays a vital role in the redox system. The usefulness of selenium is limited by its cytotoxicity and durability. When synthesized into nanoparticles, cytotoxicity can be reduced and stabilized. SeNPs induce apoptosis by generating ROS and arresting the cell cycle. ROS production is responsible for stimulating TNF-α, which could be a major problem in producing psoriatic lesions. Inflammation-related illnesses can be effectively treated with SeNPs. Lowering inflammatory mediators like IFN-γ, IL-1ß, IL-12, IL-6, and IL-2 is the key mechanism behind the anti-inflammatory action. The use of SeNPs topical gel reduced the incidence of psoriasis significantly. SeNPs have anti-proliferative and anti-inflammatory properties that limit epidermal hyperplasia. SeNPs might be a possible therapeutic approach to manage psoriasis; one of the benefits of SeNPs is their biological inertness, making them more relevant in psoriasis with fewer side effects [32].

**Fig. 4:**
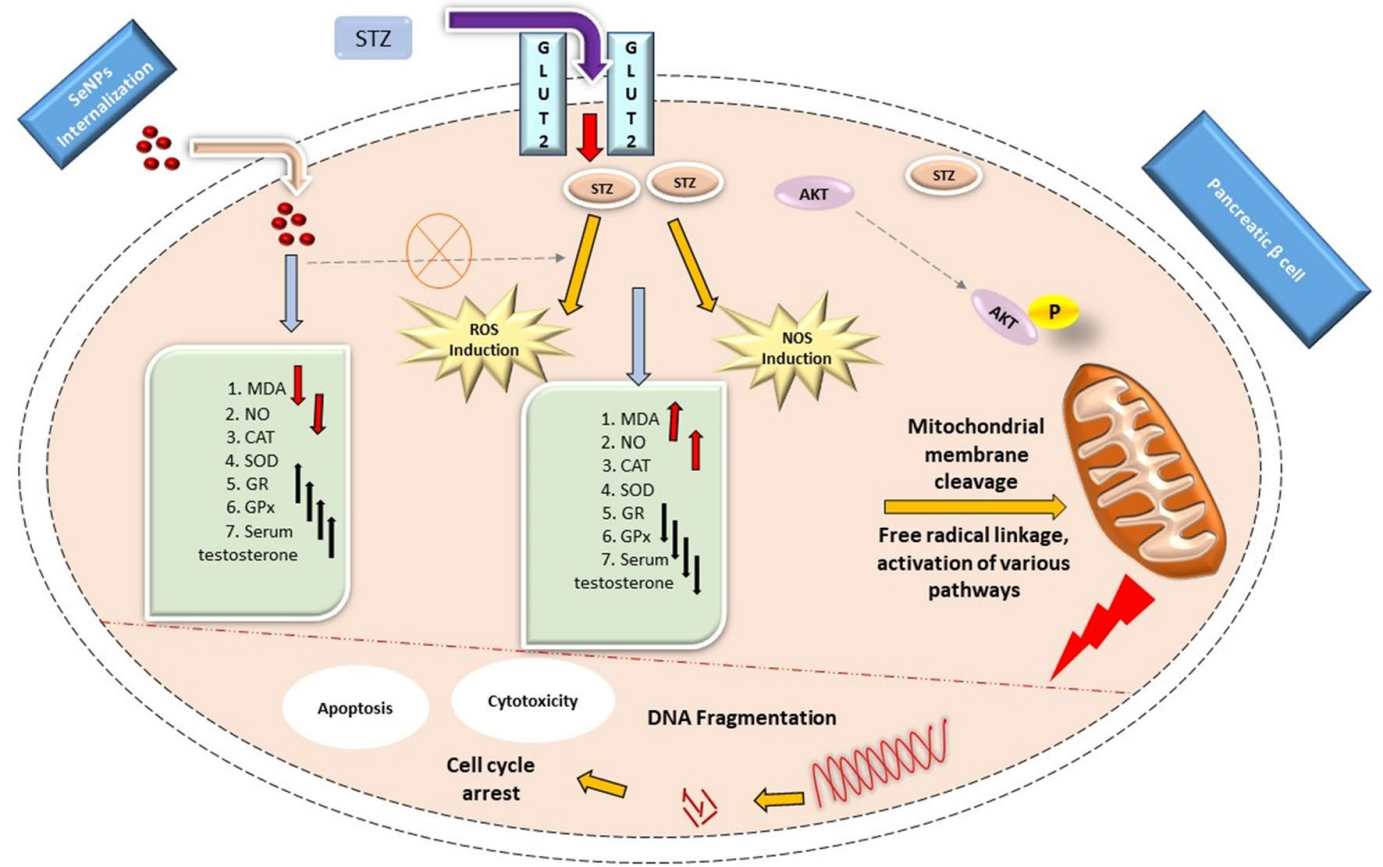
The mechanism of action and role of SeNPs in the management of STZ-induced diabetes [49]

The demonstration of the mechanism of SeNPs in psoriasis treatment is depicted in Fig. 4. Although the concentration of selenium in patients with psoriasis has been lower than average, research on its function in the disease’s etiology is limited. Selenium can affect immune reaction by altering cytokine and receptor activity or enabling immune cells to be more sensitive to peroxidation. Selenium intake was found to have an antagonistic effect on TNF- levels in patients with psoriasis. Selenium analogs have also been shown to suppress mRNA in human keratinocytes, preventing the in vitro production of UVB-induced proinflammatory cytokines [33].

#### Asthma

People living with bronchial asthma experience oxidative stress, which is unsurprising given the chronic disease’s aggressive character. Se is considered a good dietary source for GSH-Px activities. Se is important in the inflammatory asthmatic response, especially when interacting with adhesion molecule activation. Endothelial cells are critically involved in the enrollment of leukocytes in inflammatory responses, and cellular adhesion molecules such as P-selectin, ICAM-1, VCAM-1, and ELAM-1 are key mediators [34].

Novel therapies that target particular compounds, often in the form of monoclonal antibodies, have been evaluated in randomized trials and even permitted for use in clinical practice and the usual therapy alternatives. Anti-IgE omalizumab, anti-IL-5 mepolizumab and reslizumab, anti-IL-5a benralizumab, and anti-IL-4a dupilumab are all examples of this. Several alternative chemicals addressing new molecular mechanisms, including selenium NPs, are being researched.

Se is a powerful dietary antioxidant beneficial to many forms of human health. Asthma is associated with higher oxidative stress, and Se consumption has been suggested to play a part in asthma etiology. However, no significant relationships between Se status and asthma prevalence have been found in human investigations. Various rodent studies suggested that the advantages of Se supplementation may vary depending on an individual’s basic Se status. It includes Th cell proliferation and mechanistic investigations that have revealed information on the impact of Se on immune cellular functions [35].

### Atherosclerosis

Se has revealed a substantial inverse relationship between Se level and heart-related disorders. Furthermore, Se administration is beneficial in preventing cardiac-related disorders in people with low initial Se levels. SeNPs and Na_2_SeO_3_ were found to have anti-atherosclerotic action in most animal tests. In an apolipoprotein E-deficient animal model of atherosclerosis, Junying Xiao et al. examined the anti-atherosclerotic effectiveness of SeNPs functionalized with chitosan (CS-SeNPs) Na2SeO3, as well as the associated processes. Epithelial NF-B stimulation and, as a result, target gene activity and adhesion molecules were inhibited by CS-SeNPs. These SeNPs reduced vascular endothelial impairment (as indicated by a rise in serum nitric oxide and a reduction in arterial adhesion molecule expression) and inflammatory processes (as evidenced by a reduction in macrophage infiltration and proinflammatory molecule expression). The current study adds to our understanding of Se’s anti-AS processes and highlights Se’s prospects as a therapeutic approach for AS [36]. The anti-atherosclerotic potential of SeNPs in apolipoprotein E defective (ApoE) mice with elevated cholesterol levels were recognized by Leilei Guo et al. The findings suggested that after a few weeks of therapy, mice treated with SeNPs or a statin individually exhibited significant alleviation from vascular damage.

SeNPs can lower bad cholesterol, triglycerides, and LDL cholesterol levels while elevating HDL cholesterol levels. SeNPs drastically decreased oxidative damage while increasing NO and GPx, SOD, and catalase activity. It also reduced H2O2-induced toxicity and oxidative stress in endothelium by raising SOD and GPx activity. These findings suggested that SeNPs may help mice with hypercholesterolemia and vascular damage by modulating lipid metabolism and decreasing peroxidation by antioxidant selenoproteins [37]. Because of their high bioavailability, significant bioactivity, and less toxicity, SeNPs have been regarded as a good choice for biomedical applications.

On the other hand, its long-term biological consequences and biosafety are unclear. In a recent report by Xiao et al., SeNPs increased oxidative stress by decreasing the activity of enzymes and the development of protective seleno-enzymes. SeNPs aggravated hyperlipidemia by causing a lipid metabolic abnormality in the liver. SeNPs were given for a long time and caused liver and renal damage. These findings provide new information about the biosafety of SeNPs and other biological nanostructures [38].

### Liver injury

SeNPs have a strong anti-inflammatory effect and the ability to control the pro-and anti-inflammatory cytokine ratio. The suppression of NF-B expression is part of a process driving this feature. This implies SeNPs’ capacity to suppress liver pro-inflammatory mediators and restore the internal structures of the treated models. Aziza B. et al. compared the effectiveness of SeNPs and its free form Se versus thioacetamide-induced liver fibrosis in mice. The findings revealed that SeNPs and Se intake reduced the action of liver enzymes, oxidative stress markers, and inflammatory mediators while increasing the activity of enzymes as compared to the thioacetamide group. The reduction of oxidative stress and inflammation could be the processes via which SeNPs or Se exert antifibrotic effects [39].

Most of the studies show that SE-NPs may have antioxidant and anti-inflammatory properties. Hossam Ebaid et al. studied the preventive properties of SeNPs toward carbon tetrachloride-induced liver damage in rats. In the CCl4-injected rats, SE-NP preprocessing dramatically reduced AST, urea, creatinine, MDA, LDH, and GSH levels to normal values. In CCl4-treated rats, SeNPs improved both liver function and hepatic tissue. SE-NPs have been shown to counteract CCl4-induced liver damage indicators and recover the antioxidant capacity to lipid profiles, hepatic tissue, and activity [40]. Adnan et al. conducted a new study to assess the production of clove buds by nanoprecipitation approach with SeNPs for the treatment of liver injury in a thioacetamide-induced model. Micronuclei proportions, genomic abnormality, and types were reduced significantly when selenium-loaded clove NPs were utilized. TNF-α and IL-6 levels in rats treated with clove extract and SeNPs were significantly lower than in rats treated with thioacetamide individually. In rats given clove nanoparticles, GPX antioxidant potential was dramatically increased. The clove-loaded SeNPs showed excellent antioxidant and scavenging capabilities, which helped to reduce thioacetamide-induced cytotoxicity and tissue injury [41].

In recent years, s-allyl glutathione (SAG), a glutathione homolog, has been studied for its antioxidative and liver-protective properties. Vennila Krishnan et al. used the medicinal herb Spermacoce hispida to synthesize selenium nanoparticles (Sh-SeNPs) that were combined with SAG (SAG-Sh-SeNPs). These SeNPs were tested in rats to see if they could protect them from acetaminophen (APAP)-induced liver and kidney damage. According to the findings, therapy with NPs preserved the liver and kidney tissue morphology. Due to the combined impact of SAG and Sh-SeNPs, SeNPs provided better protection versus APAP toxicity than Sh-SeNPs. These conjugated SeNPs prevented the liver from APAP intoxication by lowering oxidative stress, increasing indigenous antioxidants, and maintaining metabolic activities, making them a good choice for overcoming APAP toxicity in liver fibrosis [42]. Because the liver enzymes grow back after treatment with the produced nanoparticles, SeNPs can be used to replace current APAP hepatic injury treatments. However, SeNPs treatment improved the histopathology of the liver [43]. Hepatotoxic consequences are possible with overdosing on paracetamol (APAP), a well-known and extensively used medication. Adel Amin et al. intended to check the hepatoprotective activity towards APAP-induced hepatic damage. Nano-Se treatment increased the hepatic antioxidant defence mechanism and reduced cellular DNA fragmentation susceptibility.

SeNPs protect from APAP-induced liver toxicity by improving liver function, reducing oxidative stress mediated by catalase, SOD, and GSH, and decreasing hepatic DNA fragmentation, a hallmark of apoptosis in the liver [44]. SeNPs and their ability to defend against autoimmune hepatitis have yet to be completely explored, and the antioxidant activities of SeNPs in hepatoprotection are unknown. Kaikai Bai et al. produced chitosan-functionalized SeNPs (CS-SeNPs), tested them for antioxidative properties and free-radical scavenging ability. In the concanavalin A (Con A)-induced liver injury mouse model, the hepatoprotective effect of SeNPs versus autoimmune liver problems was also investigated. The oxygen radical absorption rate of CS-SeNPs was adequate, and they could scavenge DPPH, superoxide anion, and peroxides. Because of its potential to enhance Se absorption, it preserved the mice against Con-A-induced peroxidation in the liver by slowing the oxidation process and increasing the functions of superoxide dismutase, glutathione peroxidase, and catalase. By improving the redox balance in the liver, SeNPs have a strong hepatoprotective ability toward Con A-induced liver injury [45].

### Diabetes

SeNPs are widely accepted as one of the alternate options for controlling deadly diabetes due to their non-toxic properties. Because of its harmful impact on pancreatic beta cells, streptozotocin, a broad-spectrum antibiotic, causes diabetes in mouse models. STZ increases the levels of MDA and NO in rats but also lowers the antioxidant capability of CAT, SOD, GR, and GPx. It interacts with glucose molecules and promotes Akt phosphorylation by using specific receptors called GLUT 2 receptors. STZ causes oxidative stress, which reduces the antioxidant ability of CAT, SOD, and other antioxidants, resulting in a decrease in testosterone levels, mitochondrial breakage, and DNA fragmentation, eventually resulting in apoptosis. Internalization of SeNPs occurs via receptor-mediated endocytosis. It reduces the generation of ROS and NOS in selected models by boosting the antioxidant capability of CAT, POD, and serum testosterone and lipid levels. SeNPs also protect the testes from histopathological destruction. SeNPs improve insulin production and cell multiplication. SeNPs’ anti-diabetes action is hypothesized to include their ability to scavenge ROS and key changes of HSP70 and SIRT1. In the hereafter, SeNPs could be a valuable and effective technique for developing nanomaterials to manage severe diabetes [46–49]. The mechanism of selenium nanoparticles in the treatment of diabetes is depicted in Fig. 4.

In recent work, Dabei et al. attempted to synthesize SeNPs from roselle plant leaf extract. Se NPs were found to be capable of enhancing the drop in blood testosterone produced by STZ-induced diabetes. The oxidative stress markers of the testicular tissue, such as NO and lipid peroxidation, were dramatically reduced by SeNPs. It also raised the glutathione concentration and antioxidant enzyme activity in testicular tissues. These findings suggested that SeNPs could help reduce oxidative stress caused by diabetes, particularly in testicular tissue [48].

In their investigation, Saleh et al. looked at the anti-hyperglycemic potential of SeNPs in diabetic rats caused by streptozotocin. Hepatic function indicators, overall lipid, cholesterol and triglycerides, LDL cholesterol levels, and glucose-6-phosphatase activity might all be reduced by SeNPs. SeNPs boosted malic enzymes, hexokinase, glucose-6-phosphate dehydrogenase activities, liver and kidney glycogen stores, and HDL cholesterol [50]. In a recent study, Amany Abdel et al. coupled metformin with chitosan functionalized nanoparticles (CTS-Se-NPs) in a rat model to see if they may influence insulin levels, liver damage, cytotoxicity, and cardiac injury markers of T2DM.

The antidiabetic efficacy of oral metformin and CTS-Se-NPs reduced the incidence of T2DM consequences, presumably by enhancing sensitivity to insulin or acting as a radical scavenger. This combination has natural antidiabetic properties. This combination treatment produced a more notable antidiabetic impact, as seen by significant reductions in morning blood glucose and insulin levels, increased anti-apoptotic gene (BCL-2) overexpression, and reduced expression of apoptotic genes [51]. Se-NPs are a unique therapy option for type 2 DM. In another study, Abdulmalek et al. examined the impact of Se-NPs alone and in conjunction with the usual antidiabetic medicine metformin in patients with STZ-induced type 2 diabetes. Their findings showed that the Induced model hurts systemic and liver tissues, resulting in severe oxidative stress, hyper-inflammation, and a disruption in the insulin signaling pathway. The amounts of functional insulin signaling proteins 3ß/pIRS1/pAKT/pGSK-pAMPK improved dramatically. SeNPs had an anti-inflammatory activity by lowering cytokine levels and restoring the equilibrium among oxidative stress and antioxidant state. Their study identified the mode of action of simultaneous SeNPs and metformin as a potential therapeutic approach that eradicates major diabetes problems, and insulin sensitivity was identified in their study [52].

### Anticancer

Low Se uptake could result in a higher cancer risk. Most of the studies proposed the effect of Se in the management of various cancers. A reduced level of SelP (selenoprotein) may be related to a greater chance of prostate, kidney, colon, and lung cancers. Being an integral part of GPx, selenium enhances the antioxidant activity of the internal redox system and decreases tissue injury, and prevents aggregation of free radicals [53]. Selenoproteins are involved in maintaining oxidative balance in cells and anticancer activity. Because of their antioxidant properties, selenium components, particularly selenoproteins, play a key role in antioxidant defense. Selenium derivatives could be utilized to treat cancer, and selenium nanoparticles have recently received much interest. Utilizing conventional treatment and radiation synergistically, particularly the ability to regulate anticancer medicines’ efficacy and severity [54].

SeNPs penetrate cells through endocytosis, which is regulated by transporters. The pH of cancerous cells is acidic, and the redox system has an imbalance. SeNPs cause oxidative stress in the endoplasmic reticulum by generating free radical damage to the mitochondrial cell membrane, resulting in the leaking of membrane proteins. The breakdown of the inner mitochondrial membrane permits proteins to seep out and activates caspases, which causes apoptosis. This cellular effective stress controls the activation of various molecular pathways, notably NF-κB, Wnt/β-catenin, MAPK/Erk, PI3K/Akt/mTOR, and apoptosis-inducing mechanisms. The NF-κB affects tissue homeostasis by signaling inflammatory and oxidative stress. Oncogenic signaling requires the MAPK/Erk, VEGF, PI3K/Akt/mTOR, and Wntβ/-catenin pathways. By modulating these pathways, SeNPs inhibit cell growth and growth-promoting signals in tumor cells. Cancer cells lose their signaling pathways as a result of SeNPs, which limits their proliferation.

**Fig. 5:**
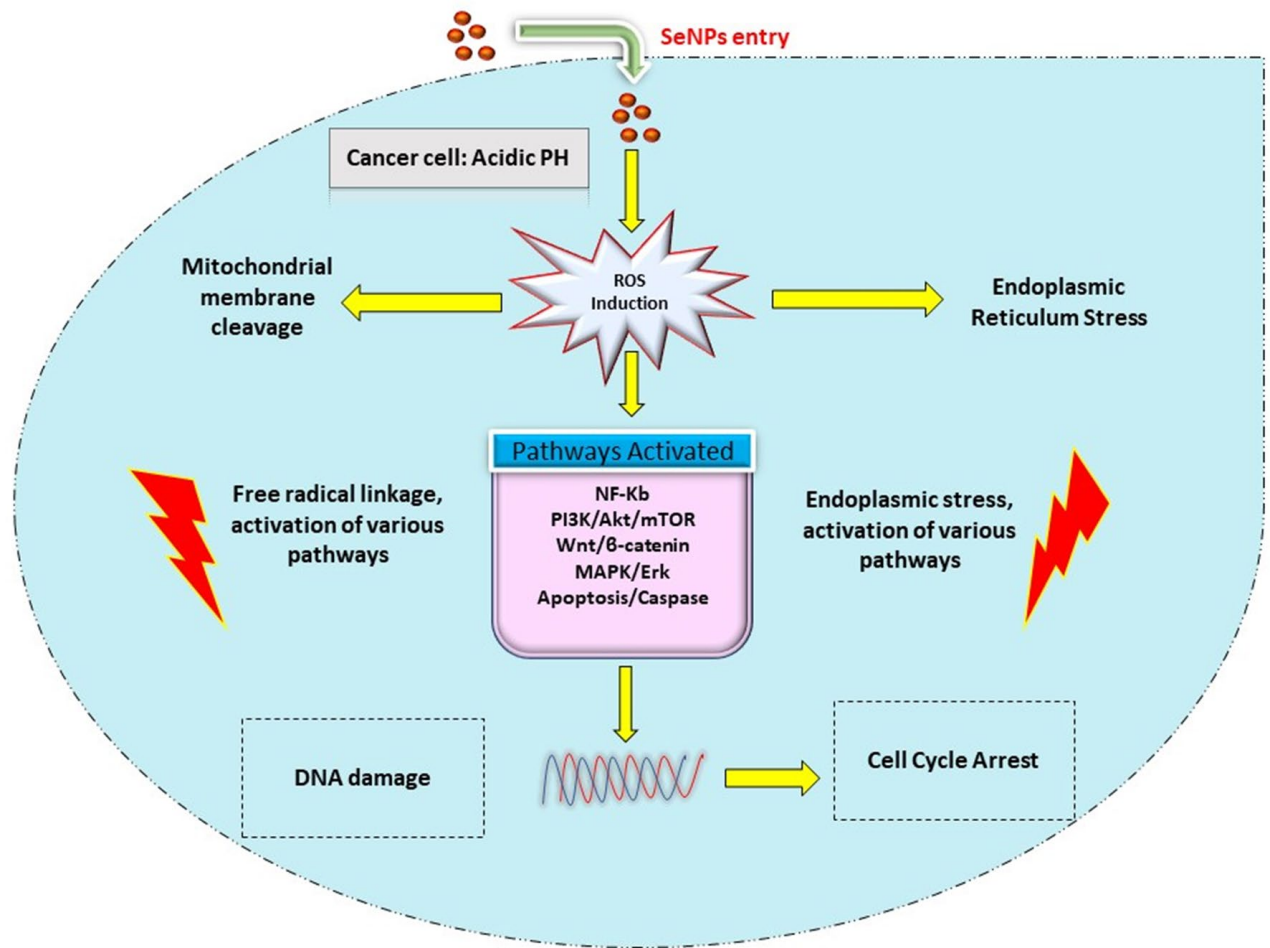
Selenium nanoparticles’ mechanism in managing cancer through different pathways [8]

Disruptive cellular processes cause DNA fragmentation, which causes cell cycle arrest (CCA), and leads to apoptosis [10]. Through caspase 9, the intrinsic pathway is activated inside the cell, resulting in oxidative stress, i.e., DNA damage. The cell-killing activity of SeNPs is through the increased synthesis of ROS that results in the destruction of DNA and ultimately results in the death of cancer cells [55]. Autophagy is the process related to the apoptosis of cells that engulfs cell organelles and eventually leads to cell killing, so any development in autophagy might be a promising approach for managing various types of cancers. In certain investigations, it is reported that SeNPs can maintain autophagy, resulting in cell apoptosis. After the treatment with SeNPs, it was reported that the internal autophagy starter Beclin-1 activated increased the expression of LC3-2 and decreased the expression of p62 on 1st day of treatment; from these, it would be concluded that SeNPs result in the production of autophagosomes and also enhance the autophagy process [56–58]. The demonstration of the mechanism of SeNPs in the management of cancer is depicted in Fig. 5.

SeNPs have already shown intensifying promise as effective therapeutic systems, particularly in cancer therapeutics, when used in conjunction with very well chemotherapeutics such as 5-fluorouracil, doxorubicin, and irinotecan, and also oligonucleotides such as siRNA, resulting in synergism and trying to overcome MDR. SeNPs could be co-delivered with certain conventional anticancer drugs and siRNA, and through their synergistic action, they can oppose MDR [59]. In general, chitosan-SeNPs were shown to have antioxidant action by decreasing ROS generation in a particular dose and increasing the survival of BABLC-3T3 cell lines. SeNPs could also incorporate anticancer medicines such as DOX, allowing controlled drug delivery in acidic endosomes and lysosomes. They have also been studied as mRNA transporters in cancer management. Because the folate receptor is typically upregulated in various cancer types, folic acid has been extensively explored as a substrate for the derivatization of NPs for the treatment of cancer. By increasing NP intake by cancer cells via receptor-mediated endocytosis, active targeting with FA has been demonstrated to improve the overall anticancer activity of these vehicles while minimizing off-target impacts in normal tissues. SeNPs that were functionalized with FA (FA-SeNPs) have been explored to treat breast and hepatocellular carcinoma [5, 60–62]. Jingxia Tian et al. analyzed the role of SeNPs combined with radiation on NSCLC A549 and NCI-H23 cell growth, penetration, translation, and apoptosis in a latest report. This incorporated SeNPs reduced the cell growth activity of the selected cell lines in a dose-dependent approach. It significantly restricted the stimulation of cell growth-related proteins and migration proteins such as MMP2 and MMP9 but stimulated the cleaved caspase-3 and cleaved caspase-9 [63].

Selenium has antitumor activity and could be useful in the treatment of osteosarcoma. According to cellular research, inorganic and organic selenium have lethal, anti-proliferative, and pro-apoptotic impacts on several osteosarcoma cell cultures. These effects could be achieved by oxidative damage induced by selenium molecules, which causes p53, proapoptotic proteins, and caspases to be activated. Therapy with selenium in rats injected with osteosarcoma decreased tumor size but did not remove cancer [64]. Luke D. et al. used a unique green process to manufacture pure naked SeNPs. These SeNPs inhibited the growth of human melanoma and glioblastoma cells.

Furthermore, SeNPs could have been employed as a protective coating for medical devices. Ultimately, at some very small doses, such “naked” SeNPs can be employed as biological agents with antimicrobial and anticancer activities [65]. In prostate cancer, SeNPs are thought to be the most effective. Some reports have shown that prostate cancer cells can be stimulated with SeNP to promote cell cycle progression and autophagy. MiR-16 has been shown to target cyclin D1 and BCL-2 to increase SeNP apoptosis deliberately. According to the findings, plasma selenium contents are favorably correlated with miR-16 patterns and are associated with total and disease-free survival time [66].

### Infectious diseases

Because of their tiny size and greater surface area, SeNPs have gained popularity in managing certain microbial infections. SeNPs’ antibacterial efficacy is significantly linked to their size, with 81 nm SeNPs being more beneficial towards MRSA than 124 nm SeNPs [67]. The common mechanism behind the antimicrobial effect of SeNPs is to oppose the proliferation of microbes through biofilm disruption. Generally, the sensitivity or resistance to any antibiotic is mostly due to barriers like the microbe’s cell membrane or cell wall, which act as their defense system for the external environment. Excessive Se ions will rupture cell walls and impair the membrane’s permeability, causing intracellular balance disruption and microbiological malfunction, resulting in microbial cell death. While modern antibiotics may effectively manage most bacterial infections, some particularly smart microorganisms or drug-resistant bacteria continue to threaten human health. Se NPs have been shown to suppress various bloodstream pathogens caused by pathogenic bacteria, making them an effective chemosensitizer for improving bacteria’s killing [68]. The antimicrobial potency of SeNPs is likely to be due to mitochondrial dysfunction caused by a decrease in the concentration of ATP levels; a rise in the internal levels of ROS, resulting in oxidative stress, resulting in loss of bacterial resistance to SeNPs, protein synthesis is inhibited and DNA mutation; and damage membrane potential and destabilization of the bacterial cell walls [5].

SeNPs, when conjugated with certain antimicrobial molecules, may exhibit an effective antimicrobial action. In humans, plants, bacteria, and phages, a molecule known as lysozyme promotes the direct killing of bacteria due to its defense mechanism involved in innate immunity. SeNPs have the potential to be an effective antibiotic option for a variety of bacteria, including dangerous multidrug-resistant pathogens. The SeNPs can significantly disrupt cell wall structure, direct bacterial eradication, or the intracellular induction of antibacterial metabolites killed by bacteria [69]. The action of SeNPs as an antiviral agent is linked to their involvement in controlling the functionality of selenoproteins, making them suitable antiviral targets with broad antiviral activity [70].

Chung et al. synthesized BSA-stabilized SeNPs with antibacterial activities against S. aureus that were fewer than 40 nm in size. The SeNPs achieved IC50 values substantially lower than human dermal fibroblasts, indicating a favorable safety margin for its effective antibacterial use [71]. SeNPs act by enhancing ROS synthesis, which triggers bacterial cell lysis. It should be functionalized for active and specific targeting of SeNPs towards bacterial cells. For this purpose, selenium is conjugated with MRSA (Se-MSNP). It exhibited potent activity against MRSA compared to SeNPs alone, which were ineffective against E. coli. In another study, the functionalization of acetylcholine and quercetin showed synergism in both MRSA and E. coli and inhibited cell multiplication.

Quercetin exhibits a bactericidal effect, and acetylcholine could increase the bioavailability of SeNPs [5, 72]. Huang et al. developed a potent compound by combining quercetin and acetylcholine to the membrane of SeNPs. Quercetin shows potent antimicrobial properties and the neurotransmitter acetylcholine, which can bind to the bacterial cell’s receptor. Compared to quercetin and acetylcholine SeNPs alone, these conjugated SeNPs showed significantly improved antibacterial effectiveness towards multidrug-resistant superbugs (MDRs). At modest doses, Qu–Ach@SeNPs are powerful towards MDRs like MRSA. These SeNPs bind to the bacteria’s cell wall and potentially cause membrane damage, resulting in a significant combinatorial antibacterial activity that suppresses MRSA [72]. El-Sayyad et al. have shown that SeNPs have antibacterial properties against various microorganisms that cause persistent urinary tract infections. The CN-SeNPs outperformed the biogenic SeNPs in antibacterial activity, suppressing both gram-positive and negative bacteria. It also prevented *E. coli* bioactivity by triggering bacterial lysis, causing cell wall rigidity and deformation. As a result, CN and SeNPs established a synergistic effect, resulting in a more potent antibacterial action against most of the microorganisms tested [73].

SeNPs have also been reported to be effective in the therapy of tuberculosis. After conjugation of Isoniazid with mannose, it exhibited synergistic actions through mannose receptors, and it promoted phagocytosis by delivering Isoniazid in acidic lysosomes. Through ROS stress and PI3K/AKT/mTOR signaling pathways, SeNPs produced apoptosis and autophagy in the infected macrophages [74]. Selenium molecules were employed as antifungals, and various investigations on SeNPs’ antifungal efficacy were conducted. Biogenic SeNPs have been shown to have antifungal properties against Candida albicans and Aspergillus fumigatus in preliminary experiments. SeNPs can inhibit CDR1 and ERG11 expression levels in Candida albicans, lowering sensitivity to antifungal treatments[75]. SeNPs might be novel therapeutic strategies with significant antimicrobial and antibiofilm capabilities that could be used in clinical situations to treat bacterial infections. In a recent study, Josheghani et al. aimed to see if SeNPs had bactericidal and antibiofilm properties toward *Vibrio cholerae* in vitro. Because of glutathione (GSH) depletion, SeNPs might extend the slight delay and significantly limit the growth rate of bacteria. Interacting with bio-SeNPs altered membrane potential and disrupted bacteria’s cell wall, releasing proteins and carbohydrates [76].

In the recent work by Zhang et al., the bio-SeNPs were developed by the Providencia sp. DCX. These bio-SeNPs had potent antibacterial properties against pathogens. Protein and polysaccharides outflow was seen due to membrane permeability alterations and cell wall breakdown. The antibacterial capabilities of the as-synthesized SeNPs aided the oxidative impairments generated by ROS. The findings revealed that various bacteria might be efficiently suppressed and damaged, implying that biosynthesized SeNPs may be used as antimicrobial agents to treat bacterial pathogenic infections [77].

SARS-CoV-2 one of the deadly virus that hit world in 2019 killed millions of people [78, 79]. SeNPs’ defensive functions are mostly achieved via their assimilation with selenoproteins. Cellular antioxidant systems containing selenoproteins, primarily GPXs, and TXNRDs, are essential for lowering peroxidation characterized by abnormal ROS caused by viral infections. Viral particles offer a potential mode of action for using Se in COVID-19 disease control and prevention. Because of its probable capacity to repair GPX and TXNRD function, minimize viral-induced cell death, defend endothelium cells, and lower blood platelet aggregation, sodium selenite has been recommended as a COVID-19 precautionary measure and immunotherapy [80]. Wang et al. demonstrated an oblique flow detection kit based on Se NPs-modified SARS-CoV-2 nucleoprotein that recognized anti-SARS-CoV-2 IgM and IgG in human plasma within 10 min using the naked eye. This study showed that the kit founded on Se NPs might recognize anti-SARS-CoV-2 IgM and IgG in human plasma and blood with ease, speed, and sensitivity, demonstrating the utility of Se NPs in both COVID-19 identification and statistical assessment [81] (Table 1).

**Table 1:** Several SeNPs’ role in treating various infectious conditions with their mechanism of action

SeNPs	Antimicrobial action	Mechanism/Effect	References
SeNPs with ketoconazole	Antifungal effect	Increased ROS stress results in decreased production of ergosterol	[82]
SeNPs with oseltamivir	Antiviral effect towards H1N1	Inhibition of caspase-3 and ROS sensitivity in host cells through p53 and AKT signalling pathways	[83]
SeNPs coated with PEI inserted with siRNA addressing the enterovirus 71 VP1 gene	Antiviral effect towards Enterovirus 71	Reduced expression of VP1 and inhibited the growth of Enterovirus 71, by blocking the Bax signalling pathway, the number of cells trapped in the sub-G1 phase was lowered.	[84]
SeNPs with atovaquone	Antiparasitic effect	Elevated the ratios of immune mediators IFN-γ, TNF-α, IL-12, and iNO, and reduced IL-10 levels.	[5]
SeNPs	Antibacterial effect against E. coli	Reduced exopolysaccharide (EPS) production inhibited the formation of biofilm.	[85]
SeNPs with RBCM and Bacteria-responsive gelatin NPs	Antibacterial effect against MRSA	Induction of ROS generation to disrupt bacterial cell membrane	[86]
SeNPs with Isoniazid	Antimycobacterial effect against Mycobacterium tuberculosis (Mtb)	Induces autophagy, apoptosis, and M1 anti-bacterial polarization, and activation of intracellular Mtb killing	[74]

### Neurodegenerative diseases (NDs)

Neurological disorders are one of the major challenges in healthcare, which mostly affects older individuals. Due to various challenges in drug delivery to the brain, mainly the blood-brain barrier (BBB) and blood-cerebrospinal fluid barrier (BCSFB) that limits the entry of therapeutic molecules. The SeNPs offer an excellent opportunity for the delivery of therapeutic molecules. NDs like Alzheimer’s disease, Parkinson’s disease, Huntington’s disease, and dementia are the most common brain disorders. The SeNPs have shown therapeutic efficacy in treating NDs compared to conventional treatments. SeNPs have a wide range of therapeutic applications [87].

A research group showed that the SeNPs have intrinsic fluorescence and diagnostic potential. Intrinsic fluorescence can be utilized to examine neuronal mechanisms. The other characteristic of SeNPs is the photoluminescence that can be used in neuroblastoma tracking and animal imaging. Some groups have shown that semiconductor monoclinic SeNPs can be used as peroxide biosensors for diagnosing oxidative stress, one of the hallmarks of NDs [87].

## SeNPs in combination with other drugs

Since NPs improve overall cellular uptake, combining them with targeted therapies may improve transportation. Most malignancies acquire multi-drug resistance and systemic toxic action, which might be mitigated by combining medications at modest doses. Several medications have also been employed with SeNPs or as SeNP conjugates. Wen Liu et al. developed 5-Flouracil (5-FU) coated SeNPs, which increased anticancer potential against the A375 cells. The surface-functionalized 5-FU exhibited apoptosis in a dose-dependent pattern due to ROS, which resulted in the expression of caspase-9 and, decreased mitochondrial membrane potential (MMP), and enhanced nuclear condensation and DNA destruction. 5FU-SeNP-induced apoptosis was related to ROS generation. This could be the potential strategy to utilize SeNPs as a conjugate for 5-FU to obtain anticancer efficiency [88].

SeNPs showed growing promise as important clinical systems, particularly in cancer therapeutics, when used in conjunction with potential chemotherapy drugs such as 5-FU, DOX, and irinotecan, and oligonucleotides such as siRNA, demonstrating synergism anticancer effects and conquering MDR [5]. In vitro and in vivo studies revealed that SeNPs, combined with irinotecan, boosted anticancer efficacy. The conjunction of Irinotecan and SeNPs influenced p53-related autophagy and partly caspase-mediated cell death in HCT-8 cells. SeNPs also boosted caspase-7, 8, and 9 functioning. SeNPs-mediated death comprises several internal and external apoptotic processes [89]. Adriamycin and SeNPs were found to be an effective cancer treatment combination. In Bel7402 hepatic cancer cell lines, this treatment showed synergistic cytotoxic action at low doses compared to individual therapies [90].

Mahsa Vahdati et al., in a report, concluded the symbiotic antibacterial property of SeNPs and lysozyme in a nanohybrid system. He investigated the tolerance of *Escherichia coli* and *Staphylococcus aureus* individually and in the nanohybrid systems. As SeNPs and Lysozyme collaborated, the nanohybrid system significantly increased bactericidal activity compared to the protein alone. The finding suggests the development of nanohybrid systems with synergistic bactericidal activities to combat antimicrobial resistance and specify important applications in biomedicine and food safety [69].

In another study, nano selenium was combined with radiotherapy to check the inhibitory effect on lung cancer. The reports found that when associated with radiotherapy, nano-Se, a novel form of Se, was discovered to have an anticancer effect, inhibiting growth, penetration, motility, and promoting death in NSCLC cells [69]. In a study, Xueyang Fang et al. developed a multipurpose nanosystem to prevent HBV-infected liver cancer. Surface-modified SeNPs with baicalin and FA targeting molecules demonstrated good bioactivity and permeability. The nano-composite demonstrated exceptional tumor cell specificity by predominantly addressing lysosomes in HepG2215 cells and utilizing caveolae-mediated endocytosis mechanisms. Furthermore, B–SeNPs–FA minimized ROS synthesis and reduced the gene expression implicated in cell growth, resulting in liver cell death. These modified SeNPs could be a novel and viable therapeutic strategy for HBV-infected liver cancer treatment [91].

### Nanoformulated SeNPs

The SeNPs have been formulated with many biological polymers. One of the studies reported that the fungal-assisted fabrication of SeNPs. Such fabrications have attracted the scientific community’s demand as a promising approach in myco-nanotecnology for therapeutic applications. Fungal myco-nanotechnology mainly focuses on matter on a nanoscale, synthesizing NPs utilizing fungal metabolites and biomass. Being a micronutrient in all organisms, especially humans, it boosts the immune responses to cancer and microbial infections. The combination of nanotechnology with the biological activity of Se will appeal to improve the physiochemical properties and enhance therapeutic applications [92].

The most important characteristics of Se are low toxicity, high bioavailability, and promising therapeutic applications; therefore, the best way to utilize the SeNPs is by mixing with polymers for the development of nano-composites having functionalities related to nanoparticles. Such nanoparticles possess the characteristics of the polymer matrix. Formulation of nano-composite polymers has significantly enhanced the properties at low concentrations of SeNP. The bio-polymers have high performance, multi-functional nano-composites, and unique characteristics such as being renewable, easily available, and environment friendly. Bio-polymers such as nano-composites are coined green composites. The green composites have been reported to have Selenium. The composites, such as fungal beta-glucans, are biodegradable, bioactive, and biocompatible, possessing immense therapeutic potential [92].

New ways of nano-formulation are being introduced, such as the development of quantum dots. One of the studies recently reported the development of selenium quantum dots (SeQDs). The SeQDs demonstrated increased delivery of drugs to BBB with multitarget effects. SeQDs are ultra-small in size and can pass the BBB hassle-free without any difficulty. The SeQDs were used in the Alzimiers animal model, showing that the animals were protected from oxidative stress, decreased in Aβ-mediated cytotoxicity prevention from the Aβ aggregation, and ultimately protected from the tau protein phosphorylation [93].

## SeNPs in drug and gene delivery

Nanoparticles exhibit unique qualities that enable the insertion of multipurpose capabilities that allow for the coupling of many therapies, resulting in more effective interventions. SeNPs are a promising drug and gene delivery approach, and their popularity has been explored in recent years. They have been used as chemotherapeutic drug carriers, for introducing genes to the site of action, and for active immunization through antigen delivery. SeNPs activate the immunological system’s cell-mediated and humoral elements, inducing proinflammatory cytokines. The generation of interferon- from splenocytes is stimulated by nanoparticles. Colloidal particles aid antigen presentation to the reticuloendothelial system. Colloidal SeNPs have been identified as extraordinary selenium analogs with chemopreventative and therapeutic capabilities. In advancing SeNPs for gene or drug delivery, surface modification (functionalization) is still vital [8]. Features like fluorescence are provided by functionalizing selenium nanoparticles using an inorganic material, which is beneficial in monitoring and imaging operations. Certain metallic and organometallic compounds have functionalized SeNPs, including Pt, Au, Ag, Rd, and iron oxide. Functionalizing selenium with ruthenium-polypyridyl and Rd-thiol complexes improved its optical characteristics for application as antiproliferative compounds and therapeutic and diagnostic vehicles [94]. One of the recent gene therapy procedures is cell cycle arrest in tumor cells by activating tumor suppressor genes and delivering pro-apoptotic genes such as p53 and PTEN.

Multiple studies have shown that when paired with therapeutic genes targeting the cell’s apoptosis mechanism, selenium’s apoptosis activation potential can slow tumor development and eliminate malignant cells. In a study, Fiona C. et al. developed the folate-targeted SeNPs and investigated their promising potential in targeted gene delivery. He intended to express that SeNPs might be used to deliver targeted pCMV-Luc DNA (pDNA) in vitro. They looked at how functionalized SeNPs expressed transgenes in different human cell lines. The SeNPs effectively bind, condense, and protect the pDNA from enzymatic hydrolysis [95]. Mesoporous SeNPs were first used in the drug delivery domain. MSe loaded with doxorubicin may synergistically boost DOX’s anticancer efficacy. These SeNPs enhanced cancer cell cytotoxicity by boosting the activities of MSe and DOX in a complementary manner [96]. Cur-loaded SeNPs have been identified to have effective antitumor potential in a murine model of Ehrlich’s ascites carcinoma, causing cell death and decreasing NF-kB signaling and EMT. Tumor-bearing mice with Se-CurNPs had a considerably lower tumor incidence and a longer mean survival rate [97]. In a study, Dhireshan Singh et al. developed SeNPs for optimal binding and targeted transport of FLuc-mRNA to hepatic cancer cells (HepG2). The findings suggest that SeNPs prevented mRNA against nuclease degradation and had low cytotoxic effects in vitro [98].

## Future prospects

Despite numerous Se supplements being accessible, Se is vital to human health. Its consumption is dependent on geographical area and the foods ingested. Se has an important role in the human body, impacting thyroid, liver, brain, and reproductive functions and possessing anticancer and microbiological characteristics. Se seems to have a restricted therapeutic window; simultaneous insufficiency and an overabundance of Se intake can produce symptoms and have been linked to a variety of disorders. SeNPs have shown promise in prophylaxis and to the management of various disorders, including diabetes, Alzheimer’s disease (AD), inflammatory disorders like RA, atherosclerosis, stroke, and antimicrobial infections.

Moreover, because elevated Se contents are more likely to produce oxidative stress in cancer cells than in healthy tissue, SeNPs are largely explored for anticancer implications. SeNPs were also loaded with various recognized chemotherapeutic agents and genetic material, exhibiting the potential to lessen the toxicity of these medications while producing synergic action. SeNPs could be derivatized to cause ROS overabundance, mitochondrial malfunction, caspase-3 upregulation, and the stimulation of the endogenous apoptotic pathway in tumor cells. SeNPs were also investigated for their ability to deliver specific siRNA, which inhibits the expression of proteins linked to tumor development and metastasis. SeNPs’ effectiveness for PTT, chemodynamic therapy, immunotherapy, and radiation synergy has also been documented in recent investigations.

Se NPs, as a type of modern nano-composites, have become very interested in dealing with the problem of antibiotic resistance. They are exhibiting much promise for new therapeutic strategies to manage infectious diseases. However, several unavoidable hurdles must be overcome before such clinical alterations occur. The most pressing issue for Se NPs throughout those trials is biocompatibility, which refers to a material’s capacity to be acceptable with biological tissue. As a result, the essential issue for the future therapeutic applications of Se NPs against various viral diseases is how to generate functional Se NPs with acceptable biocompatibility and degrading properties. The specific killing actions of Se NPs towards potential infections are the elements of their excellent anti-infection capabilities that have received the greatest attention.

Moreover, as is well known, defense mechanisms for preventing infection are among the most critical challenges in infectious disorders. Selenium’s immunity-regulating actions are assumed to be linked to selenoproteins, which play important roles in metabolic pathways and immunity. However, more research is needed to see how selenium from SeNPs impacts immunological responses by altering selenoprotein expression. Furthermore, determining the dynamics of several selenoproteins in SeNPs or inorganic sources is critical to improving our interpretation of SeNPs’ therapeutic actions.

Natural compounds, which frequently suffer from poor bioavailability despite having appealing pharmacological efficacies, have had their drug delivery properties of SeNPs exploited sparingly. Multipurpose SeNPs could be developed with greater tumor piercing utilizing tumor-penetrating peptides like iRGD, better absorption by lactoferrin derivatization, and concurrent drug entrapment and theranostic characteristics. Exploring controlled-release microparticulate formulations for encapsulating SeNPs, which might also optimize the toxicity and pharmacological profile, might be fascinating. Furthermore, investigations on the association of SeNPs with genes and chromosomes are needed, as is research on potential geno stability. The SeNPs have several limitations. Some forms are very toxic, and the amorphous SeNPS are unstable and converted into nonbioactive/gray elemental Se due to aggregation, impairing its anticancer and antioxidant activity. Such limitations should be overcome by stabilizing the SeNPs with rosmarinic acid and glucan addition [99].

Advancements in new-generation technologies like artificial intelligence or machine learning provide new, efficient solutions to existing challenges. The synthesis of SeNPs has huge challenges which have promising solutions in artificial intelligence. The NPs synthesis is influenced by several reaction conditions and physiochemical properties that determine their surface chemistry, size, and shape. There is a need for precise control of reaction conditions that influence the NP size, shape, and surface chemistry. Currently, methods used in preparing NPs are microreactors, which are laborious and time-consuming. Artificial intelligence applications might help mitigate the challenges posed [100].

.

## Conclusion

Se nanostructures, a vital trace component needed as a transcription factor for different enzymes, have emerged as a powerful tool in therapeutic applications for a range of diseases, including bacterial, fungal, and viral infections, inflammation, autoimmune disorders, neurological diseases, diabetes, and drug-induced toxicity, among others. SeNPs might have a considerable significance in most of the research findings. In this review, we emphasized the relevance of SeNPs above their elemental analogs. Hyperglycemia, colitis, and autoimmune disorders are only a few conditions in which SeNPs have shown their effectiveness. Drug-induced toxicities and genotoxicity have both been found to be efficiently combated by SeNPs. SeNPs have been discovered to influence inflammatory conditions and diabetes by inhibiting various regulatory pathways. Due to Se’s greater toxicity potential, there is still far to go in determining the therapeutic index. As a result, significant preclinical safety investigations are required before SeNPs see the light of the day. Its dose and biochemical form influence selenium’s therapeutic efficacy, and selenium nanomaterials are a good example of this due to their superiority over other selenium forms, their health hazard, strong pharmacological activities, optical and chemical characteristics that have made their way towards the use in cancer treatment and diagnosis. The aggregation of SeNPs impairs the antioxidant and anticancer activity. The toxicity and instability of the amorphous SeNPs need to be rectified using various stabilizing agents. The application of selenium as a transport mechanism is indeed an emerging and mostly untouched topic, particularly in the areas of gene delivery, also with the majority of research recently focusing on the dual handover of chemotherapeutic agents and siRNA. The mode of action and synergistic effects of selenium when combined with other therapeutic products are still unknown, and more research is needed.

## Data Availability

Data sharing does not apply to this article as no datasets were generated or analyzed during the current study.

## Abbreviations

AIM2: Interferon-inducible protein AIM2
Akt: Protein kinase B
ALT: alanine aminotransferase
APAP: Acetaminophen
APC: Apoptosis-associated speck-like protein containing CARD
ARE: Antioxidant response element
AST: Aspartate transaminase
Bax: Bcl-2-associated X protein
Bcl2: B cell lymphoma 2
BSA: bovine serum albumin
CAT: catalase
CRP: C-reactive protein
CCA: Cell cycle arrest
CI: Cellular infiltration
CI-NK cells: Cytokine-Induced Killer cells
CD: Crohn’s disease
CDK: of cyclin-dependent kinases
CP: Cyclic peptide
DPPH: 2,2-diphenyl-1-picrylhydrazyl
ECM: Extracellular matrix
EGF: epidermal growth factor
ELAM-1: Endothelial leukocyte adhesion molecule
ENA: Epithelial neutrophil-activating protein
FA: Folic acid
GPx: Glutathione peroxidase
GSH: Glutathione
HA: Hyaluronic acid
HCT-8: Human ileocecal adenocarcinoma
HDL: high-density lipoprotein
HepG2: Hepatic cancer cells
HO-1: Heme oxygenases
HUVECs: human umbilical vein endothelial cells
IBD: inflammatory bowel disease
ICAM-1: Intercellular adhesion molecule 1
ICAM-1: Intracellular adhesion molecule
IEB: Intestinal epithelial barrier
IFN-γ: interferon-gamma
ILs: Interleukins
IMQ: Imiquimod
iNOS: inducible nitric oxide synthase
KGF: Keratinocyte growth factor
LDH: Lactate dehydrogenase
LDL: low-density lipoprotein
LPO: Lipid peroxidation
MadCAM-1: mucosal addressin cell adhesion molecule 1
MAPK: Mitogen-activated protein kinase
MCP-1: Monocyte chemotactic protein 1
MDA: malondialdehyde
MDR: Multidrug resistance
MEFV: Mediterranean fever gene 16p13
MHC: Major histocompatibility complex
MMP: Mitochondrial membrane potential
MRO: Mannose-rich oligosaccharides
MRSA: methicillin-resistant Staphylococcus aureus
mTOR: Mammalian target of rapamycin
NASH: non-alcoholic steatohepatitis
NK cells: Natural killer cells
NLR: Nod-like receptors
NLRP-3: NOD-like receptor pyrin domain-containing
NO: Nitric oxide
NOD2: nucleotide-binding oligomerization domain 2
NPs: Nanoparticles
NQO-1: NAD(P)H quinone oxidoreductase 1
Nrf2: Nuclear factor-erythroid factor 2-related factor 2
NSCLC: Non-small cell lung cancer
PAMPs: Pathogen-associated molecular patterns
p-ATM: Phosphorylated ataxia-telangiectasia mutated
PGC-1α: Peroxisome proliferator-activated receptor-γ coactivator-1a
PGs: Prodigiosins
PHGPx: Phospholipid hydroperoxide glutathione peroxidase
PLGA: poly D, L-lactic-co-glycolic acid
POD: Peroxidase
PU: polyurethane
PVC: polyvinyl chloride
PTEN: Phosphatase, and TENsin homolog deleted on chromosome 10
PVP: polyvinyl-pyrrolidone
ROS: Reactive oxygen species
SAG: s-allyl glutathione
Se: Selenium
SeNPs: Selenium nanoparticles
siRNA: Single stranded RNA
SIRT1: Silent Information Regulator 1
SOD: Superoxide dismutase
STAT-4: signal transducers and transcription 4
STZ: Streptozocin
T2DM: type 2 diabetes mellitus
TEWL: trans-epidermal water loss
Th cells: T helper cells
TLRs: Toll-like receptors
TNBS: trinitro benzene sulfonic acid
TNF-α: Tumor necrosis factorα
TRx: Thioredoxin reductase
ULP: Ulva Lactuca polysaccharide
VCAM-1: Vascular cell adhesion molecule
VEGF: Vascular endothelial growth factor
